# Enigmatic Cases of Finger Proximal Interphalangeal Joint Swelling: Case Series and Focused Review of the Literature

**DOI:** 10.3390/jcm14165916

**Published:** 2025-08-21

**Authors:** Gershon Zinger, Yaakov Applbaum, Amos Peyser

**Affiliations:** 1 Department of Orthopedic Surgery, Shaare Zedek Medical Center, Faculty of Medicine, Hebrew University of Jerusalem, Jerusalem 9103102, Israel; amosp@szmc.org.il; 2 Department of Radiology, Shaare Zedek Medical Center, Faculty of Medicine, Hebrew University of Jerusalem, Jerusalem 9103102, Israel; applbaum@szmc.org.il

**Keywords:** PIP swelling, joint swelling, psoriatic

## Abstract

**Objectives:** This case series and focused literature review address the diagnostic challenges and management strategies for patients presenting with atraumatic, isolated swelling, and stiffness of a single finger’s proximal interphalangeal (PIP) joint. The dual emphasis is on synthesizing the lessons learned from our case series and providing a structured framework for clinical reasoning, including imaging, biopsy, and laboratory assessment. Non-traumatic causes of finger swelling may include rheumatological disease, atypical infection, tumor, or metabolic conditions. Routine evaluation includes history, examination, and radiographs. Additional evaluation may include ultrasound, inflammatory screening labs, magnetic resonance imaging (MRI) and computed tomography (CT). Despite these efforts, a diagnosis may still be elusive. The objective of this study is to provide a useful clinical differential diagnosis and provide lessons learned from this unique group of patients. **Methods:** Starting in February 2017, patients with isolated unilateral PIP swelling were followed. Clinical information was collected, including history, examination, and laboratory and imaging studies. Patients were followed until a specific diagnosis was obtained. In addition to the series of patients, a focused literature review is given to present additional unusual causes of atraumatic isolated PIP swelling that were not found in this series. **Results:** There were five cases that met the criteria. This includes two cases ultimately diagnosed with psoriatic arthritis, and one case each of osteoid osteoma, gout and palindromic rheumatoid arthritis. **Conclusions:** The single most effective test that helped to reach the final diagnosis in this series was the CT scan.

## 1. Introduction

Patients who present with isolated unilateral finger proximal interphalangeal (PIP) swelling can be a diagnostic dilemma. Non-traumatic causes of finger swelling in general include rheumatological disease, atypical infection, tumor, or metabolic conditions. Routine evaluation includes history, examination, and radiographs. Additional evaluation may include ultrasound, inflammatory screening labs, and magnetic resonance imaging (MRI). When the described evaluation has been carried out and still no diagnosis has been reached, both the patient and the physician are typically frustrated.

We present a combined case series and focused review of the literature to clarify the diagnostic approach to single digit PIP joint swelling. Patients who presented with swelling and stiffness of a single proximal interphalangeal (PIP) joint without a diagnosis form the basis of our report. Many had previously consulted other hand surgeons and undergone therapy or anti-inflammatory medication—both without sustained benefit. The literature review yielded limited guidance. Our aim is both to present our case series and summarize the relevant literature on monoarticular PIP inflammation, intending to offer clinicians a practical diagnostic and therapeutic pathway.

The patients in this series were followed, sometimes for years, until a diagnosis was ultimately identified. This manuscript reflects lessons learned from this series of patients. The literature review was carried out to provide other etiologies of monoarticular PIP inflammation and provide guidance on both diagnosis and treatment. We hope this combination helps the clinician to diagnose and treat patients with a complaint of atraumatic isolated PIP swelling and stiffness.

## 2. Materials and Methods

Starting in February 2017 until September 2023, patients with isolated unilateral PIP swelling were followed. We included only patients with no history of trauma and with no known inflammatory conditions. Clinical information was collected, including history and examination, and laboratory and imaging studies. The patients were asked to participate in this review and all agreed. If the final diagnosis was not identified while they were being followed in clinic, they were called periodically for additional information regarding any further evaluation and their clinical status. Each patient gave verbal consent that was documented in their chart to be included in this review.

At the initial evaluation, a history was obtained, and examination was performed. Routine evaluation included radiographs of the digit in two planes, PA and lateral. In nearly all of the patients, additional evaluation included laboratory studies for an underlying inflammatory condition (Table 1). Human Leukocyte Antigen B27 (HLA-B27) was not evaluated in these patients. Since these patients were relatively young and with apparent joint inflammation, most were sent for a formal rheumatology consultation.

Since the radiographs were almost entirely normal, additional imaging included an MRI scan to evaluate the soft tissue. Since the MRI tests in most of the patients were nonspecific, showing synovitis or tenosynovitis, most patients were also sent for a computed tomography (CT) scan with reconstructions.

An extensive review of the literature was made, evaluating the causes and diagnoses of unilateral PIP swelling. After review of the articles, the relevant references were added. In addition, focused and review articles were added that were relevant to specific diagnoses identified in the initial search.

## 3. Results

Five patients were identified who presented with unilateral PIP swelling and pain and an ultimate diagnosis was reached. These five cases are presented here, followed by a literature review. Table 2 provides a summary of the results with details of each case that follows. None of the patients had a history of an inflammatory condition. In addition, none of the patients had a family member with a known inflammatory condition or had exposure from travel to Lyme disease-infected areas. Neither of the two patients diagnosed with psoriatic arthropathy had a personal or family history of psoriasis.

**Figure 1:**
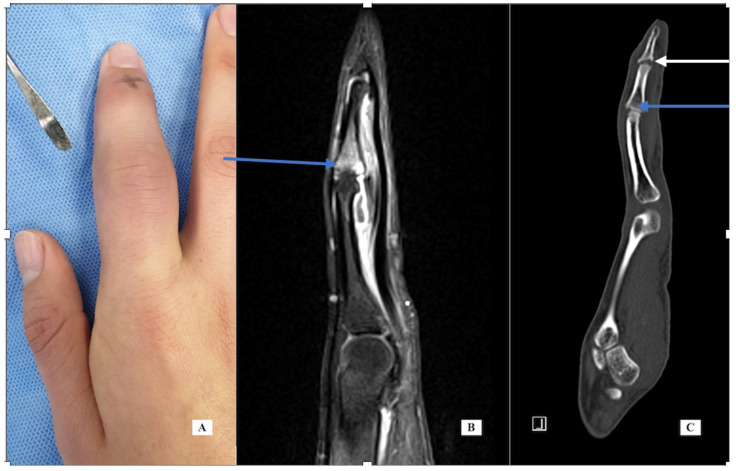
Case 1—clinical photograph of the enlarged PIP joint (**A**); MRI of the finger, sagittal view (**B**) demonstrating bone marrow edema at the base of P2; CT scan of the finger (**C**) demonstrating central erosions at the DIP joint (white arrow) and dorsal erosion at the PIP joint (blue arrow).

Case 1: A 22-year-old woman presented September 2018 with a 3-month history of right index finger swelling and stiffness localized to the PIP joint. There was no history of trauma and no other painful or inflamed joints. She had tried non-steroid anti-inflammatory drug (NSAID) medication, natural remedies, and occupational therapy, with no improvement. Her exam was notable for the swelling of the proximal phalanx and the PIP joint (Figure 1). She had mild PIP joint stiffness with flexion limited to 95 degrees (versus 105 degrees left F2). All labs for routine inflammatory screening (Table 1), X-rays and ultrasound were normal. She was sent for an MRI scan in October 2018 (Figure 1 center, post-contrast T1 fat suppressed) that demonstrated flexor tenosynovitis, joint synovitis and bone marrow edema. The report noted that “infection could not be ruled out”. She was taken to surgery (November 2018) with exploration under wide-awake local anesthesia no tourniquet (WALANT) for incisional biopsy of the thickened tenosynovium, both superficial and deep to the FDP tendon. The material was noted to be fibrotic and opaque in appearance. The material was sent for pathology, as well as cultures that included atypical mycobacterium. A specimen was sent for polymerase chain reaction (PCR—16S rDNA) testing. All the tests and cultures from surgery were negative, with pathology showing “nonspecific mild chronic inflammation” with a suggestion that “further clinical correlation is needed”. She was sent for a formal rheumatology evaluation and based on her presentation, psoriatic arthritis was suspected, and she was started on Sulfasalazine, which she took for 3 months without any improvement, so she elected to discontinue the medication. She adds that she had had no skin lesions in the past or on examination that would be consistent with psoriasis. Repeat labs for an underlying inflammatory disorder were carried out and again found to be normal. Repeat X-rays were carried out of her finger in January 2019, 6 months after the initial X-rays that showed soft tissue swelling, mild joint narrowing, and thick periosteal reaction involving the distal proximal and middle phalanges. Because there was still no improvement and no definitive diagnosis, a CT scan was performed. Her CT images from June 2021 (Figure 1, right) showed erosive changes at the PIP and DIP joints consistent with psoriatic arthritis. The CT findings were helpful in establishing a diagnosis and guiding future therapeutic options including starting biologics.

**Figure 2:**
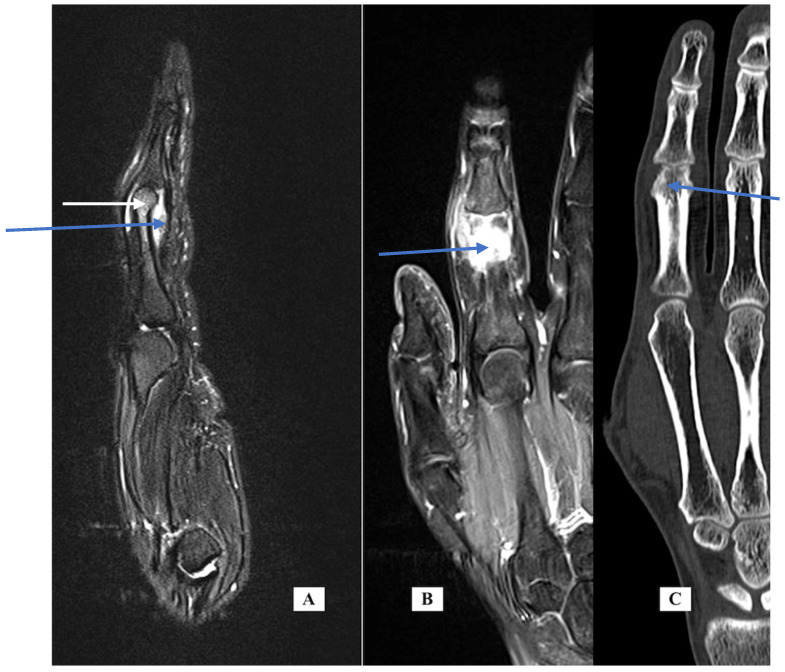
Case 2—MRI of the finger, sagittal view (**A**) demonstrating synovitis (blue arrow) and bone marrow edema of distal P1 (white arrow); coronal view (**B**); CT scan of the finger, coronal view (**C**) demonstrating marginal erosion at the radial collateral ligament origin.

Case 2: A 30-year-old female was seen in February 2019 with left index finger pain, swelling and stiffness for one year. Her exam was notable for left index finger PIP joint swelling and reduced flexion. Her history was significant for fibromyalgia diagnosed by rheumatology. An MRI of the finger (Figure 2 left and center, T1 fat suppressed with IV contrast) shows bone marrow edema of the distal half of the proximal phalanx and mild reactive changes of the PIP joint with dorsal and volar synovitis but no tenosynovitis. The report added that “infection cannot be ruled out”. A biopsy was carried out under WALANT in June 2019. The incision was dorsal over the PIP joint using the interval between the central slip and the lateral band. An incisional biopsy of the thickened dorsal capsule was sent for pathology and cultures. Pathology showed nonspecific synovitis with mild inflammation and vascular infiltration. The cultures were all negative, including fungal and mycobacteria. After the final negative culture result, the frozen biopsy specimen was sent for PCR (16S rDNA) testing and was also negative. Additional testing by rheumatology included additional labs for inflammatory markers, Lyme serology and sexually transmitted disease (STD), all of which were negative. Infectious disease recommended additional serologic markers for Q fever, Brucella and Bartonella, all of which were negative. Because of her persistent complaints of pain and stiffness, CT was recommended. The CT scan showed marginal erosions of the index P1 distally at the origin of the radial collateral ligament (Figure 2, right). This is indicative of an underlying inflammatory condition, most likely psoriatic arthritis. She was referred to rheumatology and started on methotrexate but did not experience relief. At the time of her last evaluation, she was switched to apremilast (30 mg twice per day), a medication specifically for psoriasis treatment.

Case 3: A 40-year-old female right hand dominant (RHD) full-time homemaker described waking up one morning with pain, swelling and stiffness of her right fifth PIP joint that worsened over the week. She was treated by her primary care physician with both a non-steroid anti-inflammatory drug (NSAID) for presumed gout and with oral antibiotics for possible infection. She stopped the NSAID but continued cephalosporin for a total of 6 weeks. Her labs were normal, including uric acid. On exam the finger was mildly swollen, tender and with reduced flexion. Her X-rays were normal. The finger gradually improved and returned to normal after 2–3 months. She was referred to a rheumatologist who diagnosed her with reactive arthritis, since one of her children was diagnosed with strep throat in the week before her finger symptoms began. She was sent for Antistreptolysin O (ASO) titer, which was elevated at 313 IU/mL (normal range < 200), consistent with recent strep infection. The swelling and stiffness ultimately resolved without specific treatment. About a year later, she developed Achilles tendinitis, with a similar onset of waking up with mild pain which progressively worsened over a week, then a gradual resolution over 2 months.

Approximately a year after the Achilles tendinitis, she woke up with mild pain in her knee which progressed to moderate swelling and erythema. Labs were elevated, including the Erythrocyte sedimentation rate and C-reactive protein (ESR and CRP) and the knee was aspirated twice, with up to 85 cc of fluid removed, described as clear and with negative cultures. She went to a different rheumatologist, who, based on her history and examination, diagnosed her with palindromic rheumatoid arthritis.

**Figure 3:**
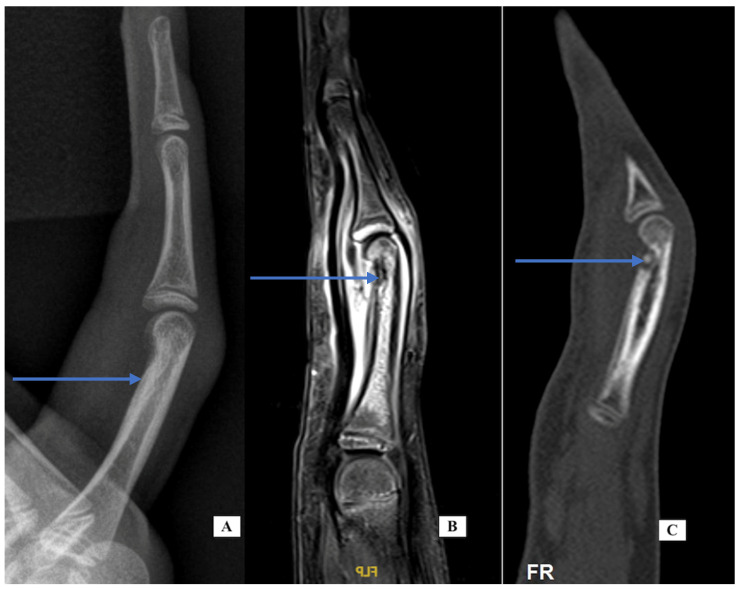
Case 4—X-ray lateral of the finger (**A**) demonstrating cortical thickening; MRI sagittal of the finger (**B**) demonstrating bone marrow edema distal P1, irregularity of the distal cortex (blue arrow) and generalized soft tissue swelling; CT sagittal of the finger (**C**) demonstrating lucency with a central calcification consistent with nidus.

Case 4: A 12-year-old female presented with a 2-month history of right F4 PIP pain and stiffness. At the time of her presentation in January 2021, she had moderate swelling of her finger, mild local warmth, no erythema, PIP motion of 25 to 70 degrees, and local tenderness. X-rays showed a periosteal reaction of the distal P1 (Figure 3, left). Labs for inflammation were normal. The MRI scan (Figure 3, center, T1 with fat suppression) showed bone marrow edema enhancement, synovitis of the PIP joint, tenosynovitis of flexor and extensor tendons, and erosion irregularity of the volar distal cortex under the P1 condyle. The report noted that the images were consistent with osteomyelitis. She was referred to a pediatric rheumatologist for evaluation. His presumed diagnosis after evaluation was inflammatory or infectious disease. He recommended additional labs including serology for hepatitis, creatine phosphokinase (CPK), anti-nuclear antibody (ANA) using immunofluorescence assay (IFA) methodology, and urinalysis, all of which were negative.

At that point, the images were reviewed with an orthopedic tumor specialist (AP), who suggested evaluation with a CT scan. The images (Figure 3, sagittal view, right) showed a small lucent cortical lesion 4 mm in length with central calcification and a periosteal reaction of the proximal phalanx consistent with osteoid osteoma (OO). She denied increased symptoms at night or pain relief with NSAIDs. She was taken to surgery using a volar approach and the lesion was treated with curettage and with local burring, but no additional adjuvant treatment. Pathology was consistent with OO and the margins were clear. Her pain resolved and the swelling and motion improved. At her last appointment, her X-rays had normalized.

**Case-specific comment:** The plain X-rays did not reveal the diagnosis that became apparent only with the CT scan. In retrospect, the periosteal reaction seen on the plain X-rays was a result of the osteoid osteoma in the distal metaphysis. Since there is no periosteum in the metaphysis, the inflammatory reaction stimulated periosteal new bone formation more proximally along the diaphysis. This periosteal new bone resorbed after removal of the nidus. Typically, the nidus is well-seen in MRI, but perhaps because of the small lesion and the resolution of the images, the diagnosis was not made via MRI but seen clearly on the CT scan.

**Figure 4:**
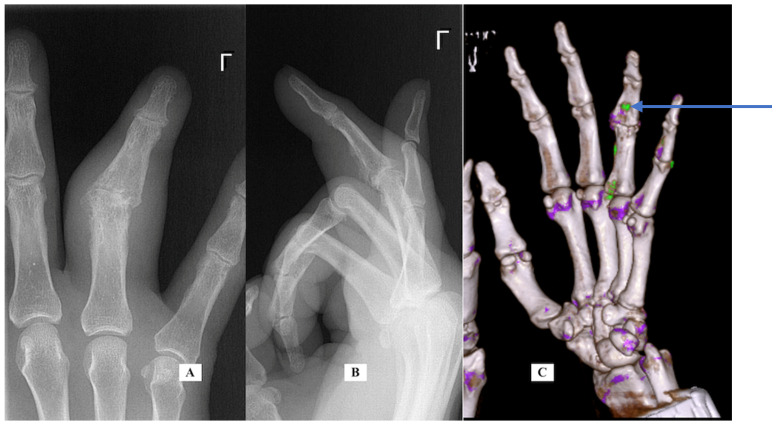
Case 5—X-ray PA (**A**) and lateral (**B**) of the finger; CT scan oblique for gout of the hand (**C**). Only the green color is significant. Purple represents calcifications.

Case 5: A 67-year-old male right-handed attorney presented with a 3-week history of left ring finger swelling of the PIP joint. He was seen elsewhere and started on both colchicine and cephalosporin without improvement. His history was significant for a fracture of the same finger as a child, a laceration of the same finger years prior and an infection 8 months before his visit, also of the same finger, that was treated with oral antibiotics and thought to have resulted from a presumed “bug bite”. He had a history of prostate cancer treated by brachytherapy. The exam was notable for moderate diffuse swelling around the PIP joint and a limited motion arc of only 5 degrees. There was no tenderness along the flexor sheath. There was mild volar and dorsal erythema. His labs were notable for mildly elevated CRP (1.4), ESR (2), and white blood cell (WBC) (14). An aspiration was performed under digital block to rule out infection and was culture negative. His X-rays (Figure 4, PA and lateral views, left and center) show angulation from the old injury and advanced PIP arthritis. CT scans for gout protocol were performed in dual-energy mode. (SOMATOM Definition Force, Siemens Medical Systems, Forchheim, Germany, with tube potentials of 80 and 140 kV with an additional tin filter). Post-processing was performed using a commercial software program (Syngo CT Workplace, Siemens Medical Systems, syngo.via VB80E) to create material-selective images, where monosodium urate (MSU) deposits were color-coded as green. A CT scan (Figure 4, right) was performed using the above gout protocol. Volar to the F4 P1 and P2 dual-energy map images demonstrate a focus of urate crystal collection (shown in green), confirming the diagnosis of gout. He was given oral indomethacin with near-immediate improvement in his pain and swelling. The finger PIP joint motion improved to an arc of 35 degrees. He was referred to his family doctor for additional treatment for his gout.

**Case-specific comment:** Despite the complex history of the digit, the most likely diagnosis was still gout based on his history and examination [1]. A CT scan with gout protocol confirmed the diagnosis.

**Summary of evaluation (Table 2):**

Laboratory screening was carried out in all patients and in one case was mildly abnormal (case 5 with gout) but was not helpful in reaching a diagnosis in any of the cases. Ultrasound was carried out in one case and showed expected inflammation and was also not helpful. A rheumatology consult was carried out in four patients and was helpful in one case, though the diagnosis with time was changed from strep exposure to palindromic rheumatoid arthritis (case 3). An MRI scan was carried out in three patients and showed significant findings, usually of a soft tissue mass with inflammatory features. CT was carried out in four patients, and all four studies were diagnostic for the final diagnosis.

Three biopsies were performed with the goal of reaching a diagnosis and ruling out chronic infection. All biopsies showed nonspecific inflammatory changes, and none were diagnostic. All the cultures were negative, including two that were sent for additional PCR testing.

**Additional uncommon etiologies for unilateral PIP swelling:**

Table 3 presents a list of different conditions that may cause unilateral PIP joint swelling. Below is a review of the more pertinent conditions that were either seen in this series or should be considered in the differential diagnosis.

Florid reactive periostitis (fibro-oseous pseudotumor):

Patient number 1 had X-rays that were initially normal. On follow-up evaluation, repeat X-rays showed reactive periostitis. The periostitis seen on the plain X-rays is suggestive of florid reactive periostitis (FRP). Gao and Zhenhua [2] describe this lesion in young adult females and warn that FRP may be confused with a malignant or infectious process. There are rare case reports involving phalanges and metacarpals [2,3]. The lesion can involve a single digit PIP joint [4]. They note that bizarre parosteal osteochondromatosis proliferation (BPOP) may also be similar in X-ray appearance depending on the stage at which the patient presents. Other diagnoses to consider with osteitis may include sapho syndrome and scarcoidosis.

Sapho Syndrome:

Sapho (synovitis, acne, pustulosis, hyperostosis and osteitis) syndrome was first described in 1987 by Chamot, a French rheumatologist [5]. The condition is better known to rheumatologists [6] but rarely reported in the radiology literature. It can involve almost any joint and can mimic infection or inflammatory arthropathy. It is typically associated with skin manifestations. A diagnosis is usually made from a combination of clinical presentation and imaging studies. Treatment is nonsurgical, with NSAIDs or other agents.

Pachydermodactyly

Pachydermodactyly (PDD) is a rare form of fibromatosis typically affecting the PIP joints of digits 2–4 [7,8]. It is typically symmetric and presents with painless and progressive soft tissue swelling. It can be associated with local trauma, including compulsive rubbing of the fingers. X-rays and ultrasounds are normal, and MRI shows soft tissue thickening. Anti-nuclear antibody (ANA) titers may be elevated and histopathology from a biopsy can confirm the diagnosis [8]. The classification includes five types [9].

Palindromic rheumatoid arthritis versus reactive rheumatoid arthritis:

Palindromic rheumatoid arthritis is typically diagnosed by a rheumatologist based on the clinical presentation of recurring flares of short duration, usually with normal laboratory findings [10]. Longitudinal studies have shown a variable progression to rheumatoid arthritis (RA). The most affected joints include the wrist, metacarpal phalangeal (MCP) and PIP joints. The condition may be seropositive or seronegative. Treatment may include NSAIDs or disease-modifying antirheumatic drugs (DMARDs) but the optimal treatment has not been identified.

Osteoid osteoma

Osteoid osteoma may be difficult to diagnose. Burger et al. [11] in 1998 describe seven cases, of which six were misdiagnosed for long periods of time, and some of the cases underwent multiple surgeries before the diagnosis was identified. Vlaic et al. [12] in 2019 describe an additional case in a child that was similarly missed.

Chronic infection:

Chronic indolent infections in the hand may also present with monoarticular PIP inflammation. Al Qattan and Helmi [13] note that chronic infections of the hand are uncommon and often the diagnosis is delayed. Cheung et al. [14] reviewed 166 cases of M. marinum tenosynovitis of the hand. Most of the patients were initially treated for an inflammatory disorder, including with NSAIDs or steroid injections. Since the initial symptoms and radiological findings are not specific, often the diagnosis is delayed. They state that MRI may show exuberant tenosynovitis, fluid around the tendons, and bone erosions. They suggest having a “proactive” approach and obtaining a biopsy for cultures and pathology. Other authors have stressed the importance of biopsy in cases where infection is suspected [15].

Psoriatic arthritis:

Psoriatic arthritis was diagnosed in two of the five patients in this series. Psoriatic arthritis was recognized as a separate entity only in 1964 by the American College of Rheumatology [16]. The condition is both progressive and destructive. Early diagnosis and treatment can prevent further progression. An active search should be made for hidden areas of psoriasis including scalp, peri-umbilical area, natal cleft (upper buttock fold), and nail abnormalities such as pitting or oncholysis. About 20% of patients with psoriatic arthritis will not have a skin lesion [17]. Treatment of psoriatic arthritis should be left to a rheumatologist, but methotrexate is often recommended as a first-line therapy [17]. Other medications such as DMARDs, anti-tumor necrosis factor (anti-TNF) agents, and biologics are used depending on response and disease severity. Inflammatory screening labs are usually normal. Imaging can be helpful but plain film changes are visible only with more advanced disease. MRI may show significant findings including enthesitis (at the capsule insertion) more often than synovitis found in RA. In addition, bone edema [18] is common. Both MRI findings are suggestive but not diagnostic of psoriatic arthritis.

## 4. Discussion

The five patients in this series were ultimately diagnosed with psoriatic arthritis (2 patients), gout, osteoid osteoma or a temporary inflammatory condition. All five patients were treated based on the specific diagnosis. The patients with psoriatic arthritis were treated by rheumatology first with methotrexate then with DMARDs after a firm diagnosis was established. The patient with gout was treated with aggressive lowering of uric acid and the patient with temporary inflammation was treated with anti-inflammatory medication. The patient with osteoid osteoma was treated with surgery. Establishing a firm diagnosis was essential to guiding treatment.

A diagnostic flow chart is provided based on the lessons learned in this series of patients and with additional diagnostic possibilities from the literature search (45) (Table 4).

A literature search for unusual causes of finger swelling will mostly identify articles describing atypical infections, including mycobacteria [15,19,20,21]. Hamard et al. [22] is one of the few articles identified that gives a differential diagnosis for unilateral finger swelling (what they term Dactylitis). When atypical infection is unlikely or already ruled out by pathology and cultures, the clinician is still left with a conundrum with regards to the further evaluation needed to reach a definitive diagnosis.

In the patients presented here with monoarticular PIP swelling and stiffness, the laboratory screening tests were all negative. When the labs and radiographs are normal, MRI seems like the next reasonable test, since the PIP enlargement with normal radiographs is soft tissue in nature and infection needs to be ruled out. This emphasis in the literature of possible latent infection, along with an MRI report that often adds “cannot rule out infection”, suggests that a culture is necessary. This combination of the literature and the MRI report encourages unnecessary open biopsy that may cause scarring and worsen stiffness. Of the five patients in this series, two were diagnosed early with a CT scan, one was diagnosed by rheumatology and the two remaining were taken to surgery, primarily to rule out latent infection. Specimens were sent for routine and mycobacteria cultures, molecular analysis and pathology.

Hamard et al. [22] describe MRI in this scenario as “fundamental” and CT is recommended only if the MRI fails to provide a diagnosis. McGonagle, Conaghan, and Emergy [18] specifically describe the MRI findings in small joints affected by psoriatic arthritis. With fat-suppressed MRI, they state that bone edema may be found at the capsule insertion and adjacent bone marrow edema. This contrasts with the synovitis or tenosynovitis seen in other inflammatory conditions. Olivieri et al. [23], in an article published after McGonagle et al. [18], directly refute their conclusions. Instead, they state that there is no evidence of enthesitis of the capsule insertion and that flexor tenosynovitis and adjacent soft tissue swelling are the MRI hallmarks of psoriatic arthritis imaging.

None of the MRI scans in this series of patients were ultimately helpful for diagnosis. While CT proved diagnostically useful in this series, MRI and other methods may have limitations due to technical factors. CT has higher special resolution than MRI and better contrast in demonstrating cortical bone changes including bony erosions, periosteal reaction, and cortical and trabecular thickening. Haugen et al. [24] note that MRI was more sensitive than X-rays in the detection of osteophytes and erosions in interphalangeal joints with osteoarthritis, but MRI had low specificity. Olivieri et al. [23] compare findings that differentiate rheumatoid compared to psoriatic arthritis. They note that in contrast to rheumatoid, psoriatic arthritis is asymmetric, involves the interphalangeal joints, and demonstrates marginal erosions. Polachek et al. [25] compare ultrasound to MRI in the diagnosis of finger joints affected by psoriatic arthritis. They find significant agreement between these modalities for the detection of inflammatory changes. Their focus was on the detection of synovitis and flexor tenosynovitis but not specifically peri-articular changes.

Simon et al. [26] use quantitative CT to investigate enthesitis in patients with cutaneous psoriasis but without any clinical or joint manifestations. They found a significant amount of enthesophytes in MCP joints compared to controls. They conclude that joint involvement precedes clinical manifestations in patients with psoriatic skin disease. In this series of patients, the CT scan was more helpful in demonstrating early periarticular erosions that lead to the specific diagnosis of psoriatic arthritis. This value of CT imaging was not expected since most of the changes were soft tissue, and MRI would have been expected to be more helpful. MRI cannot differentiate between synovitis or tenosynovitis related to infection and those of an inflammatory process. Our threshold is higher now for open biopsy and culture and we prefer a more conservative approach with CT evaluation and clinical monitoring.

CT vs. MRI vs. Biopsy in Isolated PIP Swelling

CT scans, MRI, and biopsy each have strengths and limitations in diagnosing isolated PIP joint pathology:

- CT: Offers high spatial resolution and is superior in visualizing cortical bone erosions, periosteal reaction, and subchondral bone changes. CT is especially advantageous for early detection of erosions in conditions such as psoriatic arthritis or gout. Pitfall: Limited sensitivity for soft tissue and synovial pathology.
- MRI: Excellent for soft tissue evaluation including synovitis, tenosynovitis, and bone marrow edema. However, its specificity can be limited—soft tissue changes may overlap between inflammatory, infectious, and neoplastic etiologies. MRI was not diagnostically conclusive in any case in our series. Pitfall: Cannot reliably distinguish infection from inflammation, expensive, and occasionally inaccessible.
- Biopsy: Offers definitive diagnoses in select cases, especially when infection, neoplasia, or atypical presentations are suspected. In our series, all biopsies showed nonspecific inflammatory changes and were not helpful diagnostically—cultures, including PCR, were negative. Pitfall: Invasive, risk of complications, low yield unless suspicion of infection or tumor is high.

Clinical scenarios and modality selection:

- Early erosive disease or metabolic bone disorders: CT preferred.
- Unclear soft tissue swelling: MRI.
- Unresolved diagnosis with suspicion of infection/neoplasm: Biopsy.

This manuscript is our effort to provide information for this troubling condition where there is currently a paucity of guidance in the literature. However, it should be stressed that there is significant selection bias with the five cases presented. We have provided the literature review to partially mitigate the effect of this bias.

Many of the patients in this series were symptomatic for years, saw multiple physicians including hand specialists and rheumatologists, and underwent blood tests, MRI, and open biopsies. The single most effective test that helped to reach the final diagnosis was the CT scan. The case series presented here, along with a review of the literature, can guide the clinician when presented with a patient complaining of stiff and painful unilateral PIP swelling.

## Figures and Tables

**Table 1:** Inflammatory screening panel.

Chemistry Complete blood count (CBC) Thyroid-stimulating hormone (TSH) Free thyroxine (T4) Erythrocyte sedimentation rate (ESR) C-reactive protein (CRP) Anti-nuclear antibody test (ANA) Rheumatoid factor (RF) Anti-citrullinated protein antibody (anti-CCP)

**Table 2:** Summary of evaluation and final diagnoses for the cases presented.

Case Number	Age	Sex	Finger	Labs ^1^	Rheum Consult	UTZ ^2^	MRI	CT	Biopsy	Final or Presumed Diagnosis
**1**	22	F	F2	Y	Y	Y	Y	Y	Y	Psoriatic arthritis
**2**	30	F	F2	Y	Y	N	Y	Y	Y	Psoriatic arthritis
**3**	40	F	F5	Y	Y	N	N	N	N	Palindromic RA
**4**	12	F	F4	Y	Y	N	Y	Y	Y	Osteoid osteoma
**5**	67	M	F4	Y	N	N	N	Y	N	Gout

^1^ Inflammatory screening labs. ^2^ Ultrasound evaluation.

**Table 3:** Conditions that can cause unilateral PIP swelling.

Rheumatologic	Infectious	Metabolic	Tumor/Pseudotumor
Psoriatic arthritis Monoarticular RA Juvenile or early-onset RA Polymyalgia rheumatica Sarcoidosis Inflammatory bowel disease Ankylosing spondylitis Lupus Scleroderma	Bacterial Mycobacteria Fungal Lyme disease Rheumatic fever Gonorrhea Syphilis	Gout/pseudogout Hemochromatosis Hemoglobinopathy Gaucher disease Sickle Cell Disease	Osteoid osteoma Florid reactive periostitis Sapho syndrome Pachydermodactyly

**Table 4:** Diagnostic flow chart for isolated atraumatic PIP swelling.

Step 1: Clinical Presentation Isolated, non-traumatic PIP swelling; Assess for pain, stiffness, erythema, warmth, and systemic symptoms. Step 2: Basic Evaluation History and physical exam; Plain radiographs (two views); Inflammatory screening panel: CBC, ESR, CRP, ANA, RF, anti-CCP, TSH, T4. Step 3: Imaging and Lab Results If radiographs and labs are normal: ○ Proceed to MRI. If radiographs show abnormal findings (erosions, periostitis): ○ Consider CT scan for further assessment. Step 4: Advanced Imaging MRI findings: ○ Nonspecific synovitis or tenosynovitis: Consider CT for bony detail. ○ Only soft tissue swelling, painless, young healthy patient: Consider pachydermodactyly. CT findings: ○ Erosions: Suggest psoriatic arthritis, rheumatoid arthritis, or chronic infection. ○ Nidus/calcification: Suggest osteoid osteoma. ○ Dual-energy CT suggests urate: Gout. Step 5: Referral and Further Management If inflammatory etiology is suspected: Refer to rheumatology. If infection is suspected (MRI suggests abscess, exposure history): Consider biopsy. If benign soft tissue condition (suspected pachydermodactyly): Avoid unnecessary immunosuppressive therapy and consider dermatology or conservative management.

## Data Availability

Additional data will be made available if requested.

## Abbreviations

The following abbreviations are used in this manuscript:

PIP	proximal interphalangeal
MRI	magnetic resonance imaging
CT	computed tomography
NSAID	non-steroid anti-inflammatory drug
WALANT	wide-awake local anesthesia no tourniquet
PCR	polymerase chain reaction
STD	sexually transmitted disease
RHD	right hand dominant
ASO	Antistreptolysin O
ESR	erythrocyte sedimentation rate
CRP	C-reactive protein
CPK	creatine phosphokinase
OO	osteoid osteoma
WBC	white blood cell
FRP	florid reactive periostitis
BPOP	bizarre parosteal osteochondromatosis proliferation
PDD	Pachydermodactyly
ANA	Anti-nuclear antibody
RA	rheumatoid arthritis
MCP	metacarpal-phalangeal
DMARD	Disease-modifying antirheumatic drugs
